# CPVT and Complete Atrio-Ventricular Block: The Flipside of the Same Coin

**DOI:** 10.3390/jcdd10030097

**Published:** 2023-02-23

**Authors:** Mattia Petrungaro, Antonio Scarà, Alessio Borrelli, Luigi Sciarra

**Affiliations:** 1 Unit of Electrophysiology, Belcolle Hospital, 01100 Viterbo, Italy; 2 Department of Clinical and Molecular Medicine, Sapienza University of Rome, 00100 Rome, Italy; 3 Unit of Cardiology and Electrophysiology, San Carlo di Nancy Hospital, 00100 Rome, Italy; 4 Department of Life, Health and Environmental Sciences, University of L’Aquila, 67100 L’Aquila, Italy

**Keywords:** CPVT, TVNS, Ryr2, sudden cardiac death, catecholaminergic polymorphic ventricular tachycardia

## Abstract

Catecholaminergic Polymorphic Ventricular Tachycardia (CPVT) is a rare electrical genetic disease characterized by ventricular polymorphic tachycardia and/or bidirectional ventricular tachycardia induced by the release of catecholamines caused by intense physical or emotional stress in structurally normal hearts. Mostly, it is caused by mutations in genes that are involved in calcium homeostasis, in particular in the gene encoding for cardiac ryanodine receptor (RyR2). Our observation is the first description of familial CPVT caused by mutation of the RyR2 gene, linked to the complete AV block.

## 1. Introduction

Catecholaminergic Polymorphic Ventricular Tachycardia (CPVT) is a rare electrical genetic disease characterized by ventricular polymorphic tachycardia and/or bidirectional ventricular tachycardia induced by the release of catecholamines caused by intense physical or emotional stress in structurally normal hearts [1]. Mostly, it is caused by mutations in two genes: one in the gene that codes for cardiac ryanodine receptor (RyR2) and causes an autosomal dominant form, and the other in the gene that encodes for the cardiac calsequestrin (CASQ2) and leads to an autosomal recessive form [2].

These genes are essential in sarcoplasmic reticulum calcium release during cardiac contraction. More recently, other gene mutations have been associated with an atypical form of CPVT, such as the CALM1 gene (encoding calmodulin) and the TRDN gene (coding for triadin), also related to calcium (Ca^2+^) homeostasis [2,3].

Catecholaminergic Polymorphic Ventricular Tachycardia is a dangerous disease because it can cause sudden death in young people. In fact, the mean age of onset of CPVT is 8 years old and more than half of patients experience syncope or cardiac arrest before the age of 20 years. The clinical manifestations of CPVT are usually prompted by adrenergic triggers, such as physical activity or emotional stress. The clinical manifestations of CPVT are usually triggered by adrenergic stimuli, such as physical activity or emotional stress. Most patients with CPVT show normal electrocardiogram, echocardiogram and cardiac magnetic resonance imaging. The exercise stress test is the most important diagnostic test, since, by determining the release of catecholamines, it can induce ventricular arrhythmias characteristic of this pathology (in particular, polymorphic ventricular tachycardias and bidirectional ventricular tachycardias). However, the diagnosis can also be made in the presence of a mutation in the genes associated with CPVT, which in the majority of cases is the RyR2 gene. However, mutation of the RyR2 gene has variable penetrance and expressivity and therefore the clinical manifestations can be heterogenic. Moreover, genetic diagnosis is essential to identify patients at risk and to implement preventive and curative treatments. Catecholaminergic polymorphic ventricular tachycardia can lead to sudden cardiac death and it is therefore important to begin drug and behavioural therapy. The most indicated drugs are non-selective beta-blockers, which may be combined with flecainide. Behavioural therapy is based mainly on avoiding situations that may cause excessive adrenergic activation [1]. ICD implantation should be considered in patients with CPVT who experience arrhythmogenic syncope and/or documented bidirectional/PVT while on the highest tolerated beta-blocker dose and on flecainide [1].

Our observation is the first description of familial CPVT caused by mutation of the RyR2, linked to the complete AV block.

## 2. The Case

**Figure 1:**
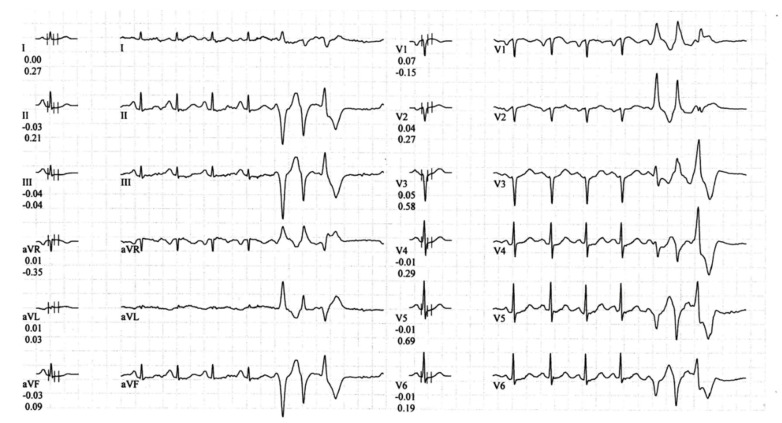
Polymorphic NSVT recorded during the stress test performed by one of the probands in our case (D.M.).

**Figure 2:**
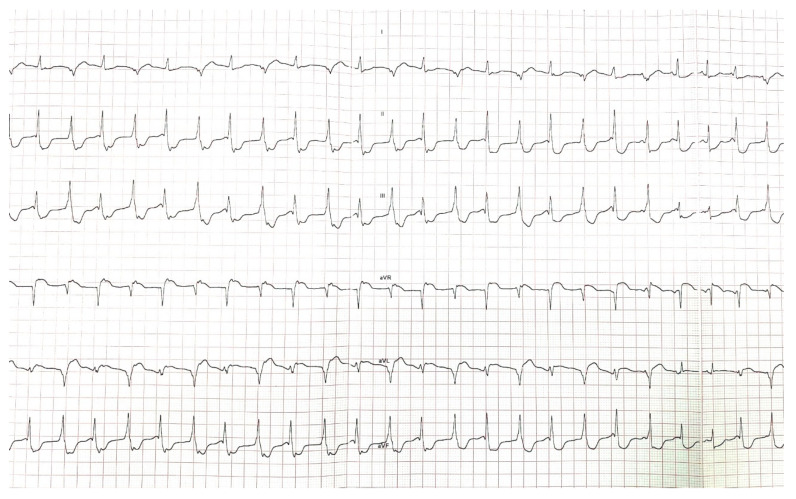

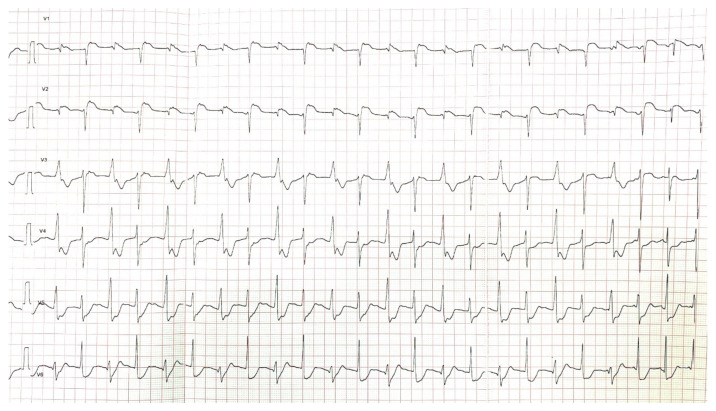
Bidirectional ventricular tachycardia recorded during the stress test performed by one of the probands in our case (D.M.).

**Figure 3:**
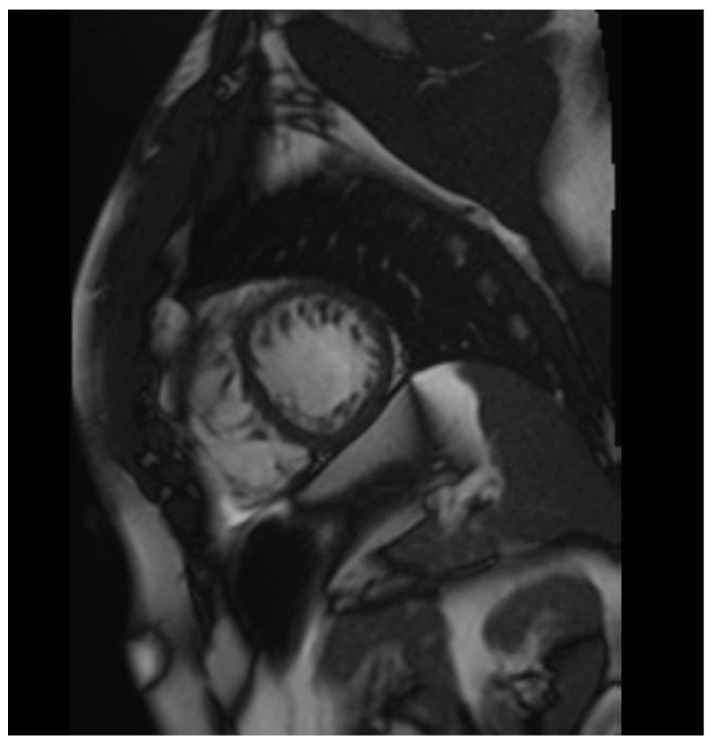
E.M. Cardiac MRI. SSFP cine sequence. Short-axis view, mid-segments. The image shows left ventricular non-compaction. MRI: magnetic resonance imaging. SSFP: steady-state free precession.

**Figure 4:**
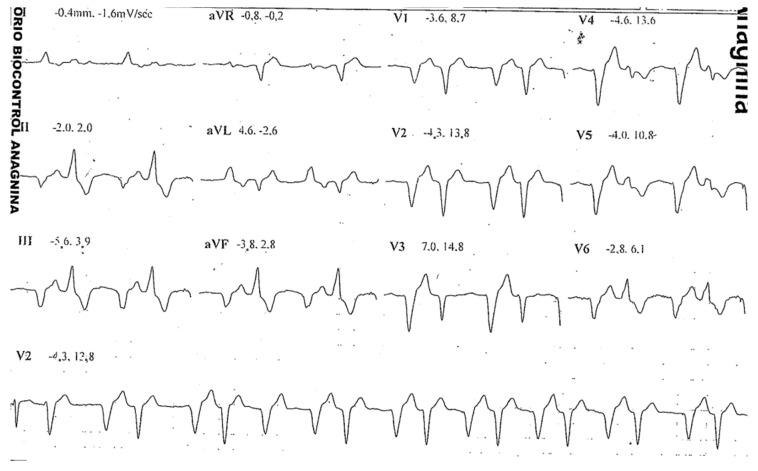
Ventricular bigeminism recorded during the stress test performed by the father (B.M.).

**Figure 5:**
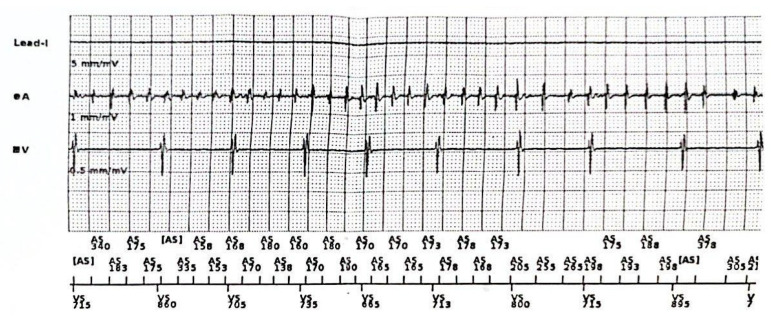
Atrial high-rate episode recorded by the pacemaker registry.

Our case series refers to an Italian family. The father (B.M.), aged 59 years, came to our attention after an implanted bicameral pacemaker four years before, at the age of 55, a relatively young age for implantation, caused by complete atrio-ventricular block. Our patient has no family history of sudden cardiac death, dilated cardiomyopathy and arrhythmias and has no siblings. His three children (E. M. female 27 years; D. M. male 25 years; B. M. female 17 years) showed evidence of polymorphic non-sustained ventricular tachycardia (NSVT) or bidirectional ventricular tachycardia during a stress test (Figure 1 and Figure 2). Therefore, they performed cardiac MRI and underwent a genetic test. All three children were found to be carriers of the same pathological microdeletion of the RyR2 gene at the genetic test according to the 2016 edition of the International System for Human Cytogenomic Nomenclature: arr[GRCH37]1q43 (237466987_237516073). An array comparative genomic hybridization (aCGH) technique was used to detect copy number changes in the RYR2 gene. The aCGH analysis of the probands identified a heterozygous deletion on chromosome 1 (1q43) that was approximately 49 kb in size, encompassing the whole of exon 3 of the RYR2 with flanking intronic regions. Genetic testing was performed using the SNP array technique with cytoSNP-850K platform at an average resolution of 100 Kb. The genetic test also included a search for mutations in the PLN, TMEM43, KCNJ2, CASQ2, CTTNNA3, DES, DSC2, DSG2, DSC2, JUP, PKP2 and LMNA genes, and the entire panel of dilated cardiomyopathy-related genes, which were, however, found to be normal, except for a mutation of uncertain significance in the JUP gene in only one of the three offspring. The three children also showed left ventricular non-compaction at cardiac magnetic resonance imaging (MRI) with a non-compacted and compacted ratio > 2.3 (Figure 3). Therefore, for this reason, both parents underwent a genetic test and cardiac MRI. Cardiac MRI was normal in both. However, at the genetic test, the father was found to be a carrier of the same genetic microdeletion as his children, whereas the mother was negative. Therefore, for the father and the three children, the diagnosis of CPVT was made. The father also performed an ergometric test (E.T.) that revealed ventricular bigeminism at the peak of the effort (Figure 4). We decided to start beta-blocker therapy with nadolol, titrating it to the maximum tolerated dose. We repeated the E.T. after 3 months, which did not result in ventricular arrhythmias at peak exercise. No major ventricular arrhythmias were recorded during follow-up, and the patient did not manifest any cardiological symptoms. However, the pacemaker registry showed a few episodes of paroxysmal atrial high-rate episodes (approximately 30 min) interpreted as probable episodes of atrial fibrillation (Figure 5). With regard to the children, the eldest daughter (E.M.) had also suffered from epilepsy from the age of 2 and a half until the age of 11. The son (D.M.) had patent foramen ovale, which closed in 2010 after a stroke. The youngest daughter (B.M.) suffered from patent foramen ovale, headaches and initial speech difficulties treated with cycles of speech therapy with resolution of the problem. All our patients carrying the RyR2 mutation, both the father and the three children, were treated pharmacologically with beta-blocker therapy titrated to the maximum tolerated dose. A non-selective beta-blocker such as nadolol was preferred. Subsequently, the efficacy of this therapy was demonstrated by repeating the exercise stress test in all our patients without wash-out from beta-blocker therapy, and no ventricular arrhythmia was recorded in any of the subjects. Finally, to date, no patient has had any episodes of resuscitated cardiac arrest or syncope with probable arrhythmic genesis. 

## 3. Discussion

**Figure 6:**
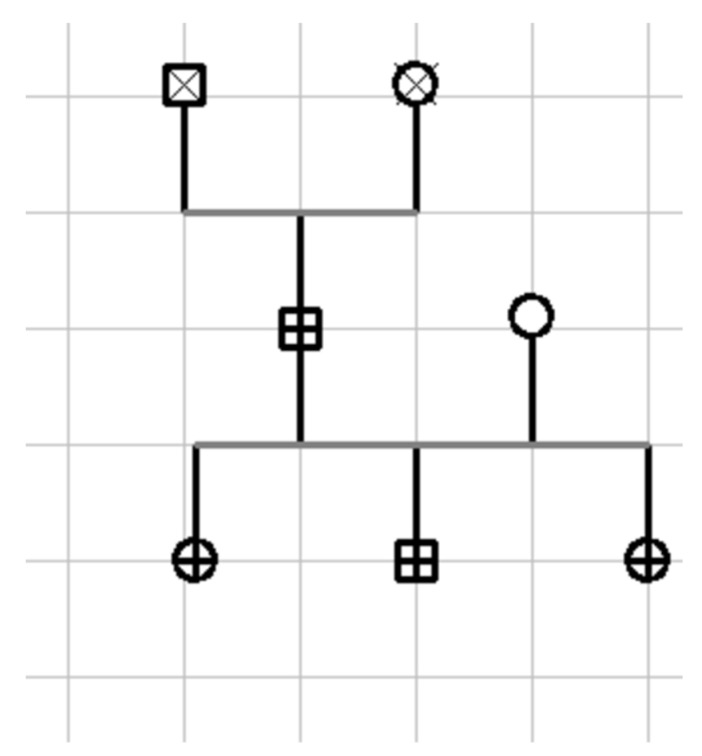
Familiar pedigree: non-carrier female: 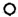; woman affected by the mutation: 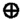; man affected by the mutation: 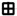; man (deceased) clinically unaffected by the mutation: 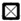; woman (deceased) clinically unaffected by the mutation: 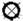.

The RyR2 gene mutation found in our case family (Figure 6), involving exon 3, has been previously reported by Ohno et al. [4], Szentpali et al. [5] and Leong IU et al. [6]. Exon 3 of the RyR2 gene encodes for a highly conserved and critical region of the RyR2 protein, and the mutation is an in-frame deletion of 35 amino acids (p.Asn57-Gly91) [6]. It should be noted, however, that the lengths of the pathological deletions of the RyR2 gene reported in the literature are shorter (1.1 kb or 3,6 kb or 37.7 kb) [4,7] than those found in our case family (49 kb).

RyR2 is an essential Ca^2+^ release channel of the sarcoplasmic reticulum and plays a central role in excitation–contraction coupling in cardiomyocytes. Abnormal Ca^2+^ leak from sarcoplasmic reticulum due to RyR2 dysfunction generates delayed afterdepolarization and can cause catecholaminergic polymorphic ventricular tachycardia in structurally normal hearts. RyR2 is predominantly expressed in both the heart and the brain, and this could be the reason why the mutation of RyR2 could have various clinical manifestations of different severity, of which CPVT is only the most dangerous. In addition, it is extremely important to take into consideration that the mutation of RyR2 has variable penetrance and expressivity. RyR2 mutation, usually caused by gain-of-function effect, causes a lowered threshold for either cytoplasmic Ca^2+^ or SR Ca^2+^ levels [8]. This increase in cytoplasmic Ca^2+^ leads to delays after depolarization (DAD) [8].

Recent data in the literature also seem to correlate the mutation of the RYR2 gene with sinoatrial node and atrioventricular node dysfunction, atrial fibrillation [9] and with some overlap syndromes, such as left ventricular non-compaction and CPVT, in particular with deletion of exon 3 of the Ryr2 genes [3,4,10,11].

In our case series, all children had non-compact myocardium at cardiac MRI unlike their father, despite having all the exact same mutation of RyR2. 

There are also data that show that a significant proportion of patients with this mutation of the RyR2 gene may have brain involvement with the presence, for example, of epilepsy and language delay [12,13].

In fact, in our case series, two of the three children had speech difficulties and epilepsy in pediatric age. 

This case is the first documented finding in the literature of an association between an RYR2 mutation and a complete atrio-ventricular block. A recent study has only shown that a RyR2 mutation could lead to sinoatrial node and atrioventricular node dysfunction [9]. However, another study [14] carried out on rats showed that the RyR2 receptor plays a role in atrio-ventricular node conduction, and that when the latter was blocked by high concentrations of ryanodine, it prolongs atrio-ventricular conduction. 

Our case series is therefore a warning to consider the RyR2 mutation in the etiology of complete AV block in a relatively young patient. Complete atrio-ventricular block was only reported in the father, while the children, although they have the same mutation, do not have conduction defects. Is it possible that they will develop it over the years? Unfortunately, this is an unanswerable question. It is probable that the varying penetrance and expressivity of the RyR2 gene mutation may result in different clinical phenotypes. As already suggested in the literature, it is also possible that the extent of the RyR2 gene deletion also influences the clinical phenotype and, in particular, the development of conduction defects [9]. However, it is crucial to monitor these patients closely over time and, on the other hand, the occurrence of idiopathic complete atrio-ventricular block in a relatively young patient may suggest investigating genetic causes. Furthermore, it can be speculated that the greater length of the RyR2 gene deletion than known in the literature may play a role in causing complete atrio-ventricular block. 

In conclusion, in consideration of our case and the recent scientific literature, we recommend performing a closer follow-up in patients with a mutation of the RyR2 gene to exclude an overlap syndrome and/or even severe alterations of cardiac conduction that may also require the implantation of a pacemaker.

Fortunately, in the current case series, no major adverse events occurred, but it is important to be extremely vigilant, especially when dealing with conditions that can cause sudden cardiac death. Therefore, in diseases with risk of sudden cardiac death, such as CPVT, it is even more important than in other diseases to make a diagnosis as early as possible because it allows the physician to initiate pharmacological or surgical treatment and to instruct the patient in preventive behavioural therapy that can also be life-saving.

In fact, if a genetic disorder had been suspected in the father when the pacemaker was implanted at the age of 55, an early diagnosis could have been made, which would have been extremely important for the children.

## Data Availability

This statement can be excluded as the study did not report any publicly archived data.
